# High-Mobility Group Box 1 Disrupts Metabolic Function with Cigarette Smoke Exposure in a Ceramide-Dependent Manner

**DOI:** 10.3390/ijms18051099

**Published:** 2017-05-20

**Authors:** Oliver J. Taylor, Mikayla O. Thatcher, Sheryl T. Carr, Jonathan L. Gibbs, Annie M. Trumbull, Mitchell E. Harrison, Duane R. Winden, Mackenzie J. Pearson, Trevor S. Tippetts, William L. Holland, Paul R. Reynolds, Benjamin T. Bikman

**Affiliations:** 1 Department of Physiology and Developmental Biology, Brigham Young University, Provo, UT 84602, USA; olivert.joseph@gmail.com (O.J.T.); mikaylathatcher@gmail.com (M.O.T.); sherylteresa@gmail.com (S.T.C.); j.gibbs93@hotmail.com (J.L.G.); annie.trumbull@gmail.com (A.M.T.); mitchellharrison@me.com (M.E.H.); paul_reynolds@byu.edu (P.R.R.); 2 College of Dental Medicine, Roseman University of Health Sciences, South Jordan, UT 84095, USA; dwinden@roseman.edu; 3 Touchstone Diabetes Center, Department of Internal Medicine, The University of Texas Southwestern Medical Center, Dallas, TX 75390; USA; Mackenzie.Pearson@utsouthwestern.edu (M.J.P.); trevorstippetts@gmail.com (T.S.T.); William.Holland@utsouthwestern.edu (W.L.H.)

**Keywords:** high-mobility group box 1 (HMGB1), ceramides, mitochondrial bioenergetics, cigarette smoke

## Abstract

We have previously found that cigarette smoke disrupts metabolic function, in part, by increasing muscle ceramide accrual. To further our understanding of this, we sought to determine the role of the cytokine high-mobility group box 1 (HMGB1), which is increased with smoke exposure, in smoke-induced muscle metabolic perturbations. To test this theory, we determined HMGB1 from lungs of human smokers, as well as from lung cells from mice exposed to cigarette smoke. We also treated cells and mice directly with HMGB1, in the presence or absence of myriocin, an inhibitor of serine palmitoyltransferase, the rate-limiting enzyme in ceramide biosynthesis. Outcomes included assessments of insulin resistance and muscle mitochondrial function. HMGB1 was significantly increased in both human lungs and rodent alveolar macrophages. Further testing revealed that HMGB1 treatment elicited a widespread increase in ceramide species and reduction in myotube mitochondrial respiration, an increase in reactive oxygen species, and reduced insulin-stimulated Akt phosphorylation. Inhibition of ceramide biosynthesis with myriocin was protective. In mice, by comparing treatments of HMGB1 injections with or without myriocin, we found that HMGB1 injections resulted in increased muscle ceramides, especially C16 and C24, which were necessary for reduced muscle mitochondrial respiration and compromised insulin and glucose tolerance. In conclusion, HMGB1 may be a necessary intermediate in the ceramide-dependent metabolic consequences of cigarette smoke exposure.

## 1. Introduction

Cigarette smoke is highly lethal, increasing age-specific mortality by roughly 300% [1]. Unfortunately, the health burden extends beyond the smoker; almost half of all children and adults are exposed to sidestream cigarette smoke, leading to hundreds of thousands of smoke-related deaths, including those from heart disease, respiratory complications, and cancers [2]. Smoke exposure, whether primary or secondary, also causes the most prevalent disorder among adults in the United States—Insulin resistance [3], which further increases the risk of virtually every chronic disease [4].

We have previously found that smoke exposure disrupts metabolic function, including insulin sensitivity and mitochondrial physiology [5,6]. Certainly, multiple mechanisms likely exist that mediate the deleterious metabolic consequences, but the sphingolipid ceramide warrants particular attention given its well-established and robust role in the etiology of metabolic disruption [4,7,8,9,10], including that arising from cigarette smoke exposure [3,6]. Importantly, ceramide accrual with smoke exposure appears to be at least partially mediated through the activation of the receptor for advanced glycation end-products (RAGE) [5,11]. RAGE becomes active with smoke exposure and, while perhaps the “original” ligand is AGE, additional signaling molecules have been identified that are likely as relevant in the context of smoke exposure. In particular, the cytokine high-mobility group box (HMGB) 1 is a RAGE ligand that is highly expressed with smoke exposure [12].

HMGB1 has previously been implicated in the pathogenesis of many inflammatory diseases, including sepsis, rheumatoid arthritis, chronic kidney disease, and more [13], all of which elicit altered states of metabolic function, including insulin resistance [14]. Based on these observations, we sought to determine the effects of smoke exposure on HMGB1 levels in skeletal muscle, the predominant insulin-responsive tissue, and whether exogenous HMGB1, via altered ceramide metabolism, is sufficient to replicate metabolic disruption evident with cigarette smoke exposure.

## 2. Results

### 2.1. Lung and Serum HMGB1 Is Elevated Following Cigarette Smoke Exposure

**Figure 1:**
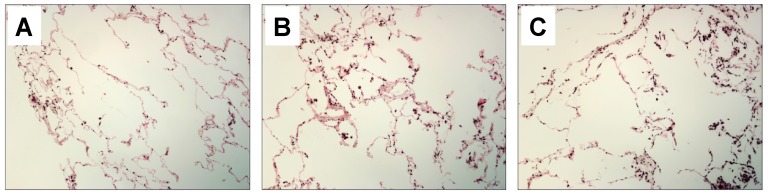
Human pulmonary biopsies from normal nonsmoker lung (**A**) (*n* = 7); smoker without diagnosed emphysema (**B**) (*n* = 15); and smoker with diagnosed emphysema (**C**) (*n* = 14) were immunohistochemically stained for high-mobility group box 1 (HMGB1). Qualitatively, HMGB1 was markedly increased in the lungs of smokers (**B**,**C**) compared to nonsmokers. Images are representative and imaged at original magnification of 63× and 4–5 random fields were evaluated by blinded individuals as to the type of sample.

**Figure 2:**
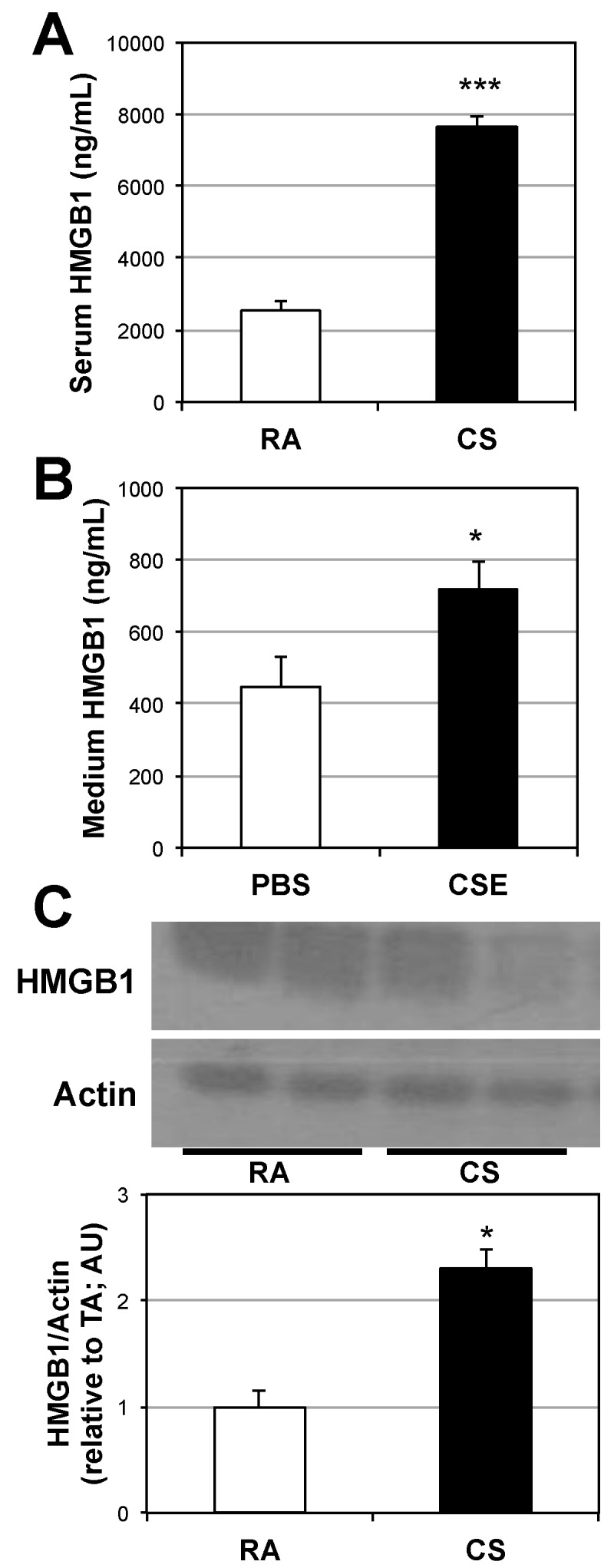
Cigarette smoke exposure increases HMGB1 in muscle. The 12–14-week-old male C57Bl6 mice were exposed to cigarette smoke (CS) or room air (RA) daily for 6 days. HMGB1 was then determined from serum (**A**) (*n* = 6); HMGB1 protein was also measured from cell culture medium of primary macrophages treated with PBS or cigarette smoke extract (CSE) (**B**) (*n* = 5) via ELISA or Western blot from alveolar macrophages from mice following smoke exposure (**C**) (*n* = 6). *** *p* < 0.001; * *p* < 0.05 for CS/CSE vs. RA.

Our initial finding was that HMGB1 protein expression is greatly increased in the lungs of chronic cigarette smokers (Figure 1). Further, we found a significant increase in HMGB1 expression in the lungs and serum of mice following 6 d of sidestream cigarette smoke exposure (Figure 2A,B). Specifically, HMGB1 expression from alveolar macrophages following smoke exposure reveals a significant increase (Figure 2B), potentially indicating the source of the increased serum HMGB1 following smoke exposure. Indeed, HMGB1 protein levels are increased in the culture medium of primary macrophages following 24-h stimulation with 10% cigarette smoke extract (CSE; Figure 2C).

### 2.2. HMGB1 Increases Myotube Ceramide Biosynthesis

**Figure 3:**
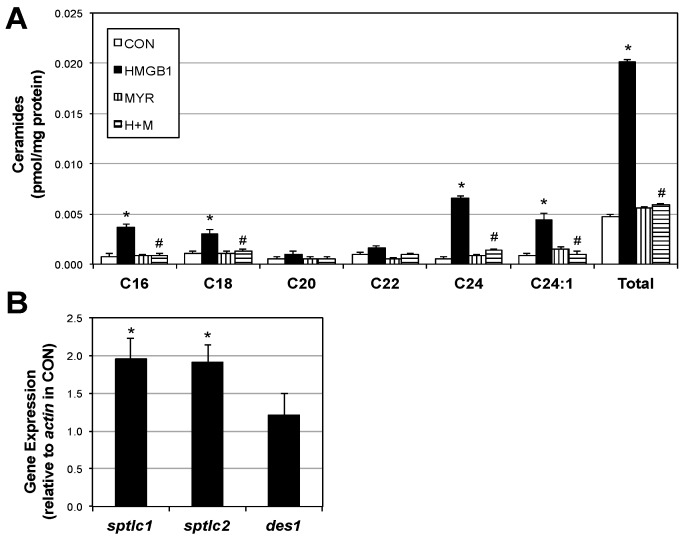
HMGB1 increases ceramide levels and biosynthetic genes in muscle cells. Following treatment (or no treatment; CON) with HMGB1 alone (H; 10 μg), HMGB1 with myriocin (H + M; 10 µM) or myriocin alone (Myr; 10 μM), murine myotubes were tested for ceramide levels (**A**) (*n* = 8); Expression of genes involved in ceramide biosynthesis was measured following HMGB1 treatment (**B**) (*n* = 4). * *p* < 0.05 vs. CON. # *p* < 0.05 vs. HMGB1.

The finding of elevated HMGB1 in muscle with smoke exposure prompted the exploration of HMGB1 itself as a mediator of metabolic differences we observed previously with smoke exposure [3,5,6]. We found that myotubes treated with HMGB1 have a roughly four-fold increase in ceramides (Figure 3A). Moreover, gene expression of two isoforms of the rate-limiting enzyme in ceramide biosynthesis, serine palmitoyltransferase (SPT) was significantly increased following HMGB1 treatment (Figure 3B). However, another biosynthetic enzyme (dihydroceramide desaturase 1; Des1) was unchanged.

### 2.3. HMGB1 Disrupts Myotube Mitochondrial Function and Insulin Signaling via Ceramides

**Figure 4:**
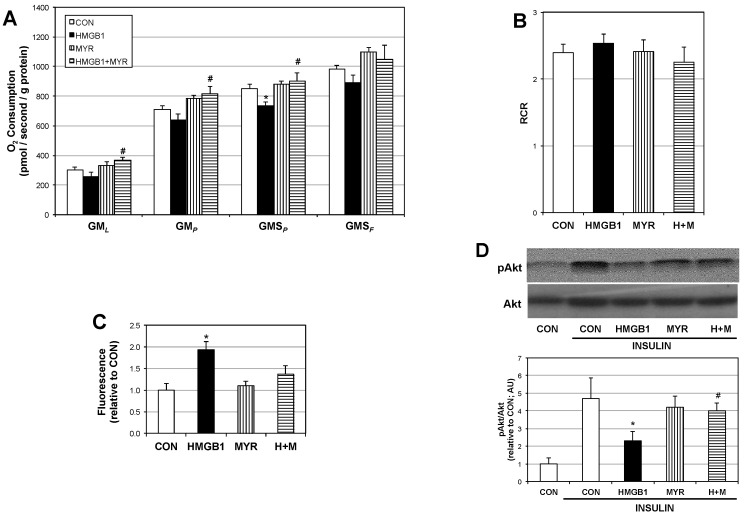
HMGB1 disrupts mitochondrial function and insulin signaling in a ceramide-dependent manner. Cells were incubated in control (CON) conditions, or with HMGB1 alone (10 μg), HMGB1 with myriocin (H + M; 10 µM) or myriocin alone (Myr; 10 μM). To measure mitochondrial respiration (**A**) (*n* = 4–5); cells were treated with: GM*_L_*: Glutamate (10 mM) + Malate (2 mM); GM*_P_*: + ADP (2.5 mM); GMS*_P_*: + Succinate (10 mM); GMS*_F_*: + FCCP (0.05 μM). Respiratory control ratio was determined by GM*_L_*/GM*_P_* (**B**) (*n* = 4–5); Production of reactive oxygen species (**C**) (*n* = 3) was determined via dichlorofluorescin (DCF) assay. * *p* < 0.05 vs. CON. Insulin signaling (**D**) (*n* = 4) via Akt phosphorylation (Ser-473) was determined by treating cells with insulin (100 nM) for 10 min prior to lysing. * *p* < 0.05 vs. CON. # *p* < 0.05 vs. HMGB1.

Myotubes treated with HMGB1 had a slight, yet significant reduction in mitochondrial respiration supported by glutamate + malate + succinate, but ceramide inhibition via myriocin co-treatment completely blocked the effect (Figure 4A), with no apparent effect on overall mitochondrial fitness (Figure 4B). Moreover, production of reactive oxygen species was almost two-fold increased over control with HMGB1 treatment (Figure 4C). HMGB1 significantly prevented insulin-stimulated Akt phosphorylation as well, though myriocin again prevented this effect (Figure 4D).

### 2.4. HMGB1 Injections Increase Muscle Ceramides and Disrupt Metabolic Function

**Figure 5:**
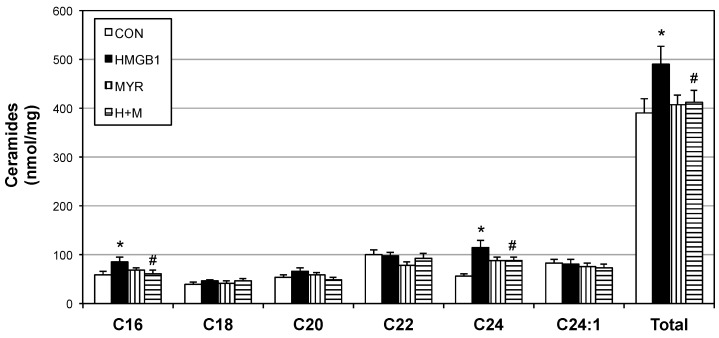
HMGB1 increases muscle ceramides. The 12–14-week old male mice received daily injections of placebo (CON), HMGB1 (1 µg/day), myriocin alone (MYR; 3 µg/kg thrice weekly), or myriocin + HMGB1 for 14 days (*n* = 6). Following the treatment period, ceramides were analyzed from soleus. * *p* < 0.05 vs. CON. # *p* < 0.05 vs. HMGB1.

**Figure 6:**
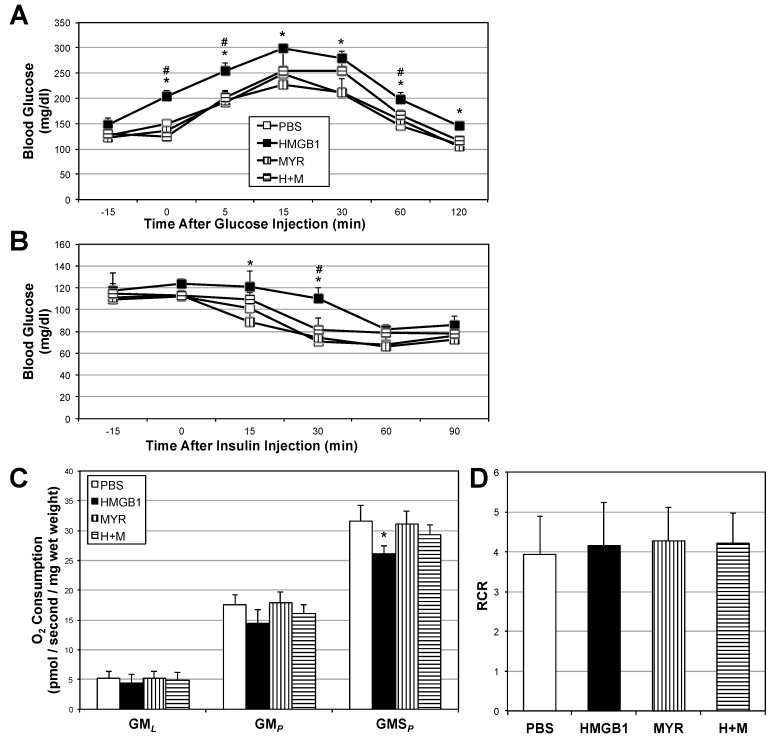
HMGB1 disrupts metabolic function in mice. Following 14 days of placebo (CON), HMGB1 (1 µg/day), myriocin alone (MYR; 3 µg/kg thrice weekly), or myriocin + HMGB1 for 14 days (*n* = 6), intraperitoneal glucose (**A**) and insulin (**B**) tolerance tests were performed on 12–14-wk old male mice (*n* = 6). Mitochondrial respiration was determined on harvested red gastrocnemius (**C**) (*n* = 6) and respiratory control ratio was determined by GM*_P_*/GM*_L_* (**D**) (*n* = 6). * *p* < 0.05 vs. CON. # *p* < 0.05 vs. HMGB1.

To explore the direct effect of HMGB1 in vivo, we analyzed the muscle and whole-body response to daily HMGB1 injections in mice. Not only were muscle ceramides significantly increased (Figure 5), but also muscle mitochondrial respiration was reduced with HMGB1 treatment (Figure 6C). Respiratory control ratio was similar among all treatments (Figure 6D). Glucose tolerance (Figure 6A) and insulin responsiveness (Figure 6B) were compromised in the HMGB1-treated mice compared with other treatments, including co-injections of HMGB1 with myriocin.

## 3. Discussion

Activation of inflammatory pathways in various tissues upsets metabolic processes. Regardless of the stimulus (e.g., visceral adipose macrophages, gut-derived LPS, etc.), one of the common consequences of inflammation is altered metabolic function, which, in some instances, leads to a loss of healthy processes. In particular, ceramide biosynthesis is triggered by numerous distinct inflammatory signals [9,15]. Importantly, we have previously shown that cigarette smoke causes metabolic disruption [3,6]; the paradigm produced from these previous studies placed ceramide as a necessary intermediate of cigarette smoke-induced metabolic disruption, however, a mechanism that signaled ceramide biosynthesis in the muscle in response to cigarette smoke exposure was unknown. The studies herein implicate the inflammatory cytokine HMGB1 as a potential lung-derived muscle ceramide inducer and subsequent metabolic disruptor in response to cigarette smoke exposure.

Previous studies of HMGB1 have centered almost exclusively on its role in the activation of inflammatory receptors, such as the receptor for advanced glycation end-products (RAGE) [16], though this effect may be relevant in multiple pathologies, including heart failure [17] and certain cancers [18,19]. Interestingly, some of the cancer-promoting effects of HMGB1 appear to be mediated through alterations in cancer cell mitochondrial bioenergetics [20]. Despite this, HMGB1 has historically been viewed through the lens of its nuclear effects, including maintenance of genome stability, replication, and repair [21], making HMGB1 relevant in diseases of overt nuclear changes, such as amyotrophic lateral sclerosis [22] and mutations giving rise to cancer [23]. Importantly, these diseases (and more) are closely associated with mitochondrial dysfunction [24], which invites speculation that HMGB1 may have a broader effect on the cell than just its role in nuclear homeostasis. Indeed, HMGB1 is known to be important in regulating mitochondrial processes in the context of autophagy [25]. However, whereas Kang et al. [20] found that HMGB1 increased pancreatic tumor cell mitochondrial action, we found that HMGB1, via increased ceramides, reduced mitochondrial respiration with the addition of succinate in muscle cells and tissue. These disparate findings may be due to cell type (e.g., pancreas vs. muscle), as well as very different experimental protocols.

Our initial findings suggest that the increased circulating HMGB1 seen with cigarette smoke exposure likely originates from alveolar macrophages before spilling into systemic circulation. These findings from rodents are supported by our observations that human lung tissue from smokers has substantially greater HMGB1 protein expression compared with nonsmokers. Importantly, RAGE is also overexpressed in the lung with cigarette smoke exposure [26]. We have previously found that RAGE is an important intermediate in the mitochondria-specific consequences of smoke exposure [5]. Furthermore, RAGE is a key receptor for HMGB1-induced pulmonary and systemic inflammation [27].

The observations from our current work extend the sphere of metabolic influence of HMGB1 beyond mitochondrial physiology to include muscle insulin signaling. Proper skeletal muscle insulin signaling is essential to maintaining whole-body insulin sensitivity. To our knowledge, we are the first to find evidence that HMGB1 expression is increased in the lung with smoke exposure and can cause insulin resistance in skeletal muscle. Moreover, the evidence suggests this may be due to HMGB1-induced ceramide biosynthesis accrual in muscle, which provides an additional context to ceramide-induced insulin resistance.

Collectively, our data indicate that HMGB1 is capable of disrupting muscle metabolic function, including mitochondrial dysfunction and insulin resistance, in a ceramide-dependent manner. These findings have obvious relevance to cigarette smoke exposure (per our model), as well as other states associated with elevated HMGB1, including injurious trauma [28], sepsis [29], oxidative stress [21], and more. Interestingly, these same insults increase ceramide accrual [9,30]. Future efforts will clarify the role of RAGE as a transducer of HMGB1-induced skeletal muscle ceramide accrual, but also the relevance of HMGB1 and/or ceramide biosynthesis inhibition as a protective and preventative therapeutic strategy in various clinical pathologies, including those habitually exposed to cigarette smoke.

## 4. Materials and Methods

### 4.1. Cell Culture

C2C12 muscle cells were maintained in DMEM + 10% FBS (growth medium; GM). For differentiation into myotubes, myoblasts were grown to confluency and the medium was replaced with DMEM + 10% horse serum (Invitrogen, Grand Island, NY, USA). Myotubes were used for experiments on day 4 of differentiation. Where indicated, cells were treated with myriocin (10 µM, Sigma, St. Louis, MO, USA) or recombinant human HMGB1 (20 nM; R&D Systems, Minneapolis, MN, USA). For insulin treatments, myotubes received 100 nM insulin (Actrapid; Novo Nordisk, Plainsboro, NJ, USA) for 10 min before harvesting. Cigarette smoke extract (CSE) was generated as previously described [8], with slight modifications. Mouse bronchial alveolar macrophages were obtained following euthanasia and exsanguination as previously outlined [11]. Seven 1-mL boluses of phosphate-buffered saline were instilled and recovered, followed by centrifugation at 1000 rpm for 10 min. After removal of supernatant, pellet was resuspended in lysis buffer and used for protein assay and Western blot, as indicated previously [31], or cultured in fresh GM. Where indicated, cells were treated with cigarette smoke extract (CSE). Briefly, one 2RF4 research cigarette (University of Kentucky, Lexington, KY, USA) was continuously smoked by connecting the filtered end of the cigarette to a vacuum pump, pulling the particles into 5 mL of DMEM-F12, and the resulting medium was defined as 100% CSE and diluted with culture medium to 10%. The total particulate matter content of 2RF4 cigarettes is 11.7 mg/cigarette, tar is 9.7 mg/cigarette, and nicotine is 0.85 mg/cigarette.

### 4.2. Animals

Male C57Bl6 mice were housed in a conventional animal house and maintained on a 12-h light–dark cycle. Animals received standard diet chow (Harlan Teklad 8604) and water ad libitum. One aspect of the study involved cigarette smoke exposure; 12–14 week old male mice were randomly divided into room air and cigarette smoke (CS)-exposed groups. Mice were placed in soft restraints and connected to the exposure tower of a nose-only exposure system (InExpose System, Scireq, Montreal, QC, Canada). Animals were nasally exposed to mainstream CS generated by research cigarettes where a computer-controlled puff was generated every minute, leading to 10 s of CS exposure followed by 50 s of fresh air. The CS-exposed group inhaled CS from two consecutive cigarettes per day for six days. Room air animals were similarly handled and restrained in fresh air for the same duration. The second aspect of the study involved HMGB1 injections. At 12–14 week of age, male mice were randomly divided into one of four groups: (1) a control group that received IP saline injections; (2) an HMGB1 group that received daily IP injections of HMGB1 (1 μg/day) [12]; (3) a myriocin group (three times a week; 0.3 mg/kg); and (4) a myriocin plus HMGB1 group. After the 2-wk course, mice underwent IP glucose (G7021; Sigma) and insulin (Actrapid; Novo Nordisk) tolerance tests. For both tests, mice were fasted for 10 hours and received an injection of either glucose (1 g/kg body weight) or insulin (0.75 U/kg body weight). Blood glucose was determined at the times indicated in the figures, using the Bayer Contour glucose meter. Tissues were harvested at the conclusion of the study period. Studies were conducted in accordance with the principles and procedures outlined in the National Institutes of Health Guide for the Care and Use of Laboratory Animals and were approved by the Institutional Animal Care and Use Committee (IACUC) at Brigham Young University (protocol #15-0201; approved 01/2017).

### 4.3. Serum and Culture Medium HMGB1 Analysis

Following the treatment period, blood was collected and allowed to clot at room temperature to obtain serum. Serum HMGB1 was measured via ELISA according to the manufacturer’s guidelines (Elabscience, Bethesda, MD, USA). To determine HMGB1 secretion from primary alveolar macrophages (obtained as described above), macrophages were treated with 10% CSE for 24 h, culture medium collected, centrifuged at 15,000 rpm to remove cells, then used directly for HMGB1 ELISA.

### 4.4. Lipid Analysis

Lipids were quantified by shotgun lipidomics using an ABI 5600+ (AB Sciex, Framingham, MA, USA), as described previously [32]. Briefly, we simultaneously identified changes in hundreds of distinct lipid species via a nonbiased approach following direct infusion of extracted lipids containing 18 mM ammonium fluoride to aid in ionization of neutral lipids and to reduce salt adducts. Data from the AB Sciex 5600+ was collected and calibrated with Analyst and PeakView Software (AB Sciex). The in-house-developed Lipid Explorer software assists with simplifying the data by identifying lipid species based on exact mass and fragmentation patterns.

### 4.5. Cell and Muscle Fiber Bundle Permeabilization

For cells, C2C12 myotubes were scraped from culture dishes with mitochondrial respiration buffer 05 (MiR05; 0.5 mM EGTA, 10 mM KH_2_PO_4_, 3 mM MgCl_2_-6H_2_O, 60 mM K-lactobionate, 20 mM HEPES, 110 mM Sucrose, 1 mg/mL fatty acid free bovine serum albumin, pH 7.1). Contents were transferred to a tube and centrifuged for 10 min at 1000 rpm at room temperature. After removal of supernatant, cells were resuspended in MiR05 plus 1 mg/mL digitonin and gently rocked at RT for 5 min before centrifugation at 1000 rpm for 5 min. After discarding supernatant, cells were then suspended in 2.2 mL warm MiR05 and transferred to chambers in the O2K Oxygraph (Oroboros Instruments, Innsbruck, Austria). Following respiration protocol (outlined below), cells were removed from the chambers and used for further analysis, including protein quantification. For skeletal muscle, red gastrocnemius was quickly removed from mice following cervical dislocation and immediately placed in ice-cold MiR05 and trimmed of connective tissue. Small fiber bundles were prepared and gently separated along their longitudinal axis to a size of 5–25 mg. Bundles were then transferred to a tube with chilled MiR05 and 50 µg/mL saponin and rocked at 4 °C for 30 min before transfer to a respirometer.

### 4.6. Mitochondrial Respiration Protocol

High-resolution O_2_ consumption was determined at 37 °C in permeabilized cells and fiber bundles with MiR05 respiration buffer as described previously (38, 47). Respiration was determined by the following substrate–uncoupler–inhibitor–titration (SUIT) protocol (32): electron flow through complex I was supported by glutamate+malate (10 and 2 mM, respectively) to determine oxygen consumption from proton leak (GM*_L_*). Following stabilization, adenosine diphosphate (ADP; 2.5 mM) was added to determine oxidative phosphorylation capacity (GM*_P_*). Succinate was added (GMS*_P_*) for complex I + II electron flow into the Q-junction. To determine full electron transport system (ETS) capacity over oxidative phosphorylation in cells, carbonyl cyanide 4-(trifluoromethoxy) phenylhydrazone (FCCP) was added (0.05 µM, followed by 0.025 µM steps until maximal O_2_ flux was reached; GMS*_F_*). Lastly, residual oxygen consumption was measured by adding antimycin A (2.5 µM) to block complex III action, effectively stopping any electron flow. This value provides a rate of respiration that is used as a baseline.

### 4.7. Oxidative Stress

Reactive oxygen species production was determined via incubation of cells with the radical-sensitive dichlorofluorescin (DCF) as previously shown [33]. Briefly, following treatment, cells were washed with warm PBS, and then incubated in 50 mM DCF in 1% BSA (Invitrogen) in DMEM (Sigma). Following a 30-min incubation, cells were washed with PBS and then Krebs–Ringer Buffer (Sigma) was added. Fluorescence was measured using the excitation/emission wavelengths 485/530 nm with a Biotek microplate reader (Biotek; Winooski, VT, USA).

### 4.8. Human Samples

Histological sections were provided by Manuel G. Cosio from the Department of Cardiac, Thoracic, and Vascular Sciences at the University of Padova, Italy. The samples were part of a previous study from 68 subjects undergoing lung surgery. Of the 68, 14 were non-cancerous smokers with severe emphysema who had lung volume reduction; 28 were smokers who had surgery for peripheral malignant nodules of which 15 did not have diagnosed emphysema; 18 were non-smokers of which 11 had surgery for lung cancers (five malignant and six benign) and seven died of accidental death (donors). The study conformed to the Declaration of Helsinki, was approved by Padova Hospital Ethical Committee (and informed written consent was obtained for each subject undergoing surgery. For the purposes of our study, five gifted lung sections from each group (non-smoker, smoker without diagnosed emphysema, and smokers with emphysema) were immunohistochemically stained for HMGB1 and 4–5 random fields were evaluated by blinded individuals as to the type of sample. Relative immunoreactivity was qualitatively assessed and representative images are shown.

### 4.9. Immunohistochemistry

For human lung HMGB1 stains, slides were blocked, incubated with primary and appropriate secondary antibodies that utilize horseradish peroxidase conjugation with the Vector Elite Kit (Vector Laboratories, Burlingame, CA, USA). Anti-HMGB1 antibodies (Abcam; Cambridge, MA, USA; Rabbit polyclonal) were employed in the experiments. No staining was observed in sections incubated without primary or secondary antibodies.

### 4.10. qRT-PCR

Total RNA was extracted and purified using TRIzol reagent (Invitrogen) according to the manufacturer’s recommendations. cDNA was synthesized from mRNA by reverse-transcriptase PCR using a commercial cDNA synthesis kit with oligo(dT) primers (iScript Select cDNA Synthesis; Bio-rad). Quantitative real-time PCR was performed with Evagreen Ssofast (Bio-Rad) using a Bio-Rad iCycler system. Primer sequences were as follows (F/R): β-actin: 5′-TGGCATTGTTACCAACTGGG/5′-GGGTCATCTTTTCACGGTTG; SPT1: 5′-TACTCAGAGACCTCCAGCTG/5′-CACCAGGGATATGCTGTCATC; SPT2: 5′-GGAGATGCTGAAGCGGAAC/5′-GTATGAGCTGCTGACAGGCA; Des1: 5′-CACCGGTACCTCGGAGCGGA/5′-GTTTGGGATTGATGAACAGGGGT. A sample containing no cDNA was used as a non-template control to verify the absence of primer dimers. β-Actin reactions were performed side by side with every sample analyzed. Changes in mRNA level of each gene for each treatment were normalized to that of the β-actin control mRNA according to Pfaffle [34].

### 4.11. Statistics

Data are presented as the mean ± SEM. Data were compared by ANOVA with Tukey’s post-hoc analysis (Graphpad Prism; La Jolla, CA, USA). Significance was set at *p* < 0.05.

## References

[1] Doll R., Peto R., Boreham J., Sutherland I. (2004). Mortality in relation to smoking: 50 years’ observations on male british doctors. BMJ.

[2] Oberg M., Jaakkola M.S., Woodward A., Peruga A., Pruss-Ustun A. (2011). Worldwide burden of disease from exposure to second-hand smoke: A retrospective analysis of data from 192 countries. Lancet.

[3] Thatcher M.O., Tippetts T.S., Nelson M.B., Swensen A.C., Winden D.R., Hansen M.E., Anderson M.C., Johnson I.E., Porter J.P., Reynolds P.R. (2014). Ceramides mediate cigarette smoke-induced metabolic disruption in mice. Am. J. Physiol. Endocrinol. Metab..

[4] Bikman B.T. (2012). A role for sphingolipids in the pathophysiology of obesity-induced inflammation. Cell. Mol. Life Sci..

[5] Nelson M.B., Swensen A.C., Winden D.R., Bodine J.S., Bikman B.T., Reynolds P.R. (2015). Cardiomyocyte mitochondrial respiration is reduced by receptor for advanced glycation end-product signaling in a ceramide-dependent manner. Am. J. Physiol. Heart Circ. Physiol..

[6] Tippetts T.S., Winden D.R., Swensen A.C., Nelson M.B., Thatcher M.O., Saito R.R., Condie T.B., Simmons K.J., Judd A.M., Reynolds P.R. (2014). Cigarette smoke increases cardiomyocyte ceramide accumulation and inhibits mitochondrial respiration. BMC Cardiovasc. Disord..

[7] Bikman B.T., Guan Y., Shui G., Siddique M.M., Holland W.L., Kim J.Y., Fabrias G., Wenk M.R., Summers S.A. (2012). Fenretinide prevents lipid-induced insulin resistance by blocking ceramide biosynthesis. J. Biol. Chem..

[8] Baeder A.C., Napa K., Richardson S.T., Taylor O.J., Andersen S.G., Wilcox S.H., Winden D.R., Reynolds P.R., Bikman B.T. (2016). Oral gingival cell cigarette smoke exposure induces muscle cell metabolic disruption. Int. J. Dent..

[9] Summers S.A. (2006). Ceramides in insulin resistance and lipotoxicity. Prog. Lipid Res..

[10] Smith M.E., Tippetts T.S., Brassfield E.S., Tucker B.J., Ockey A., Swensen A.C., Anthonymuthu T.S., Washburn T.D., Kane D.A., Prince J.T. (2013). Mitochondrial fission mediates ceramide-induced metabolic disruption in skeletal muscle. Biochem. J..

[11] Robinson A.B., Johnson K.D., Bennion B.G., Reynolds P.R. (2012). Rage signaling by alveolar macrophages influences tobacco smoke-induced inflammation. Am. J. Physiol. Lung Cell. Mol. Physiol..

[12] Reynolds P.R., Kasteler S.D., Cosio M.G., Sturrock A., Huecksteadt T., Hoidal J.R. (2008). Rage: Developmental expression and positive feedback regulation by EGR-1 during cigarette smoke exposure in pulmonary epithelial cells. Am. J. Physiol. Lung Cell. Mol. Physiol..

[13] Musumeci D., Roviello G.N., Montesarchio D. (2014). An overview on HMGB1 inhibitors as potential therapeutic agents in HMGB1-related pathologies. Pharmacol. Ther..

[14] Carlson G.L. (2003). Insulin resistance in sepsis. Br. J. Surg..

[15] Holland W.L., Bikman B.T., Wang L.P., Yuguang G., Sargent K.M., Bulchand S., Knotts T.A., Shui G., Clegg D.J., Wenk M.R. (2011). Lipid-induced insulin resistance mediated by the proinflammatory receptor tlr4 requires saturated fatty acid-induced ceramide biosynthesis in mice. J. Clin. Investig..

[16] Rauvala H., Rouhiainen A. (2007). Rage as a receptor of HMGB1 (amphoterin): Roles in health and disease. Curr. Mol. Med..

[17] Volz H.C., Kaya Z., Katus H.A., Andrassy M. (2010). The role of HMGB1/RAGE in inflammatory cardiomyopathy. Semin. Thromb. Hemost..

[18] Lin L., Zhong K., Sun Z., Wu G., Ding G. (2012). Receptor for advanced glycation end products (RAGE) partially mediates HMGB1-ERKs activation in clear cell renal cell carcinoma. J. Cancer Res. Clin. Oncol..

[19] Chen R.C., Yi P.P., Zhou R.R., Xiao M.F., Huang Z.B., Tang D.L., Huang Y., Fan X.G. (2014). The role of HMGB1-RAGE axis in migration and invasion of hepatocellular carcinoma cell lines. Mol. Cell. Biochem..

[20] Kang R., Tang D., Schapiro N.E., Loux T., Livesey K.M., Billiar T.R., Wang H., Van Houten B., Lotze M.T., Zeh H.J. (2014). The HMGB1/RAGE inflammatory pathway promotes pancreatic tumor growth by regulating mitochondrial bioenergetics. Oncogene.

[21] Tang D., Kang R., Zeh H.J., Lotze M.T. (2011). High-mobility group box 1, oxidative stress, and disease. Antioxid. Redox Signal..

[22] Lo Coco D., Veglianese P., Allievi E., Bendotti C. (2007). Distribution and cellular localization of high mobility group box protein 1 (HMGB1) in the spinal cord of a transgenic mouse model of als. Neurosci. Lett..

[23] Tang D., Kang R., Cheh C.W., Livesey K.M., Liang X., Schapiro N.E., Benschop R., Sparvero L.J., Amoscato A.A., Tracey K.J. (2010). HMGB1 release and redox regulates autophagy and apoptosis in cancer cells. Oncogene.

[24] Poyton R.O., McEwen J.E. (1996). Crosstalk between nuclear and mitochondrial genomes. Annu. Rev. Biochem..

[25] Tang D., Kang R., Livesey K.M., Kroemer G., Billiar T.R., van Houten B., Zeh H.J., Lotze M.T. (2011). High-mobility group box 1 is essential for mitochondrial quality control. Cell Metab..

[26] Chen L., Wang T., Guo L., Shen Y., Yang T., Wan C., Liao Z., Xu D., Wen F. (2014). Overexpression of rage contributes to cigarette smoke-induced nitric oxide generation in copd. Lung.

[27] Rao N.V., Argyle B., Xu X., Reynolds P.R., Walenga J.M., Prechel M., Prestwich G.D., MacArthur R.B., Walters B.B., Hoidal J.R. (2010). Low anticoagulant heparin targets multiple sites of inflammation, suppresses heparin-induced thrombocytopenia, and inhibits interaction of rage with its ligands. Am. J. Phys. Cell Phys..

[28] Peltz E.D., Moore E.E., Eckels P.C., Damle S.S., Tsuruta Y., Johnson J.L., Sauaia A., Silliman C.C., Banerjee A., Abraham E. (2009). HMGB1 is markedly elevated within 6 hours of mechanical trauma in humans. Shock.

[29] Wang H., Yang H., Tracey K.J. (2004). Extracellular role of HMGB1 in inflammation and sepsis. J. Intern. Med..

[30] Bikman B.T., Summers S.A. (2011). Ceramides as modulators of cellular and whole-body metabolism. J. Clin. Investig..

[31] Hansen M.E., Tippetts T.S., Anderson M.C., Holub Z.E., Moulton E.R., Swensen A.C., Prince J.T., Bikman B.T. (2014). Insulin increases ceramide synthesis in skeletal muscle. J. Diabetes Res..

[32] Xia J.Y., Holland W.L., Kusminski C.M., Sun K., Sharma A.X., Pearson M.J., Sifuentes A.J., McDonald J.G., Gordillo R., Scherer P.E. (2015). Targeted induction of ceramide degradation leads to improved systemic metabolism and reduced hepatic steatosis. Cell Metab..

[33] Hansen M.E., Simmons K.J., Tippetts T.S., Thatcher M.O., Saito R.R., Hubbard S.T., Trumbull A.M., Parker B.A., Taylor O.J., Bikman B.T. (2015). Lipopolysaccharide disrupts mitochondrial physiology in skeletal muscle via disparate effects on sphingolipid metabolism. Shock.

[34] Pfaffl M.W. (2001). A new mathematical model for relative quantification in real-time RT-PCR. Nucleic Acids Res..

